# Distinct osteoclastogenic potential of granulocyte-macrophage colony-stimulating factor-induced monocyte subsets

**DOI:** 10.1016/j.bonr.2025.101886

**Published:** 2025-11-10

**Authors:** Shiho Kinoshita, Yasuhiro Omata, Kojiro Sato

**Affiliations:** a Division of Rheumatology and Clinical Immunology, Department of Medicine, Jichi Medical University, 3311-1 Yakushiji, Shimotsuke-shi, Tochigi, 329-0498, Japan

**Keywords:** Osteoclast, Rheumatoid arthritis, GM-CSF, RANKL, TNF-α

## Abstract

**Background:**

Rheumatoid arthritis (RA) is a chronic autoimmune disease characterized by synovial inflammation and progressive bone destruction. Although osteoclasts mediate bone resorption in RA, recent evidence suggests that inflammatory osteoclasts differ from physiological osteoclasts in various aspects, including their progenitor origins. This study aimed to compare the osteoclastogenic potential of monocyte-derived dendritic cells (moDCs) and other granulocyte-macrophage colony-stimulating factor (GM-CSF)-induced cells to identify potential progenitor populations involved in inflammatory bone damage.

**Methods:**

Classical monocytes were isolated from human peripheral blood mononuclear cells and cultured under four conditions: (i) M-CSF, (ii) GM-CSF, (iii) GM-CSF + interleukin (IL)-4, and (iv) GM-CSF + tumor necrosis factor-alpha (TNF-α). Subsequently, to induce osteoclast differentiation, the cells were cultured with M-CSF and receptor activator of NF-κB ligand (RANKL) with or without the presence of GM-CSF, IL-4, and TNF-α, followed by evaluations via tartrate-resistant acid phosphatase (TRAP) staining and pit formation assays.

**Results:**

Cells cultured with M-CSF, GM-CSF, and GM-CSF + TNF-α differentiated into TRAP-positive multinucleated osteoclasts with bone-resorbing activity. In contrast, moDCs (condition iii) exhibited minimal osteoclast differentiation without any bone-resorbing activity. Introduction of an intermediate M-CSF culture step induces adhesion of moDCs and partially induces osteoclastogenesis. However, their differentiation efficiency and bone resorption capacity remained inferior to those under other conditions. Notably, IL-4 and GM-CSF, but not TNF-α, suppressed osteoclast differentiation.

**Conclusions:**

moDCs exhibit limited potential as osteoclast precursors under inflammatory conditions. Comparatively, GM-CSF (+ TNF-α)-induced progenitors represent a more viable inflammatory osteoclast precursor population. Overall, our results provide insights into osteoclast heterogeneity and RA-associated bone destruction mechanisms.

## Highlights

• Monocyte-derived dendritic cells (moDCs) show limited bone-resorbing activity in vitro.
• GM-CSF (+/− TNF-α)-induced macrophages exhibit robust osteoclastogenic potential.
• IL-4 and GM-CSF, but not TNF-α, substantially inhibit RANKL-induced osteoclastogenesis.

## Graphical abstract

Classical monocytes differentiate into osteoclasts upon stimulation with M-CSF followed by M-CSF and RANKL. GM-CSF, especially in combination with TNF-α, promotes the generation of osteoclast precursors. Monocyte-derived dendritic cells (moDCs) do not differentiate into osteoclasts unless pre-cultured with M-CSF prior to RANKL exposure. IL-4 and GM-CSF each inhibit osteoclast differentiation when applied concurrently with M-CSF/RANKL stimulation.

**Figure:**
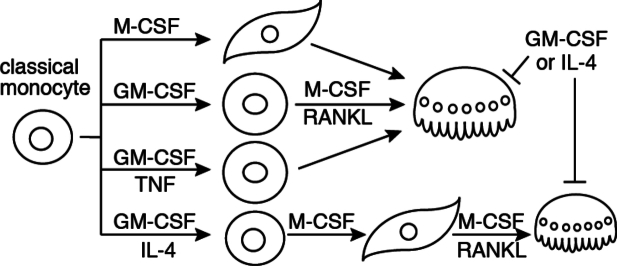

## 1 Introduction

Rheumatoid arthritis (RA) is a chronic autoimmune disease primarily characterized by polyarthritis. RA inflammation primarily affects the synovial membranes of joints; however, the precise mechanisms remain unclear, with no definitive cure currently available. Uncontrolled inflammation leads to joint deformities and bone destruction, significantly impairing the patient's quality of life. Osteoclasts play a crucial role in bone destruction. These multinucleated cells, derived from the monocyte/macrophage lineage, are the only known cells capable of resorbing the bone matrix. Therefore, elucidation of the underlying osteoclast differentiation mechanisms is of significant clinical importance (Shim et al., 2018).

Osteoclastogenesis requires two essential cytokines: macrophage colony-stimulating factor (M-CSF), which supports monocyte/macrophage survival, and receptor activator of NF-κB ligand (RANKL), a key osteoclast differentiation factor. In mouse in vitro models, bone marrow cells cultured with M-CSF are widely used as osteoclast progenitor cells (Sato et al., 2006). However, owing to the limited availability of human bone marrow cells, monocytes isolated from peripheral blood mononuclear cells (PBMCs) are used as standard progenitor sources in humans. Komano et al. demonstrated that among human monocytes, CD16^−^ monocytes exhibit a higher propensity to differentiate into osteoclasts than CD16^+^ monocytes (Komano et al., 2006). The former corresponds to the classical monocyte (CD14^+^CD16^−^) subset among the classical, non-classical, and intermediate monocyte subsets. In vitro, progenitor cells from mice and humans cultured with M-CSF and RANKL generate tartrate-resistant acid phosphatase (TRAP)-positive multinucleated cells with bone-resorbing activity, which are generally considered osteoclasts.

Emerging evidence suggests, however, that the osteoclasts involved in RA pathology fundamentally differ from the physiological osteoclasts. For instance, bisphosphonates, which inhibit osteoclast differentiation, are widely used to treat osteoporosis, but are less effective in preventing bone destruction in RA (Peris et al., 2021). In contrast, tumor necrosis factor (TNF) inhibitors in combination with methotrexate effectively prevent bone destruction in RA (Klareskog et al., 2004). These reports indicated that “inflammatory osteoclasts,” which are distinct from physiological osteoclasts, may play a central role in RA-associated bone damage. Consistently, various studies in mouse models (Yokota et al., 2014; O'Brien et al., 2016) and human cells (Yokota et al., 2021) have shown that TNF-α and interleukin-6 (IL-6), rather than RANKL, induce osteoclastogenesis.

In addition, Hasegawa et al. at Osaka University identified a novel subset, arthritis-associated osteoclastogenic macrophages (AtoMs), in the inflamed synovial membranes of collagen-induced arthritis model mice (Hasegawa et al., 2019). These cells exhibit osteoclastogenic potential in response to RANKL, particularly in the presence of TNF-α, suggesting the involvement of a unique inflammatory precursor population that may also be present in human RA. Similarly, investigators at the University of Occupational and Environmental Health discovered that human monocyte-derived dendritic cells (moDCs) generated by culturing monocytes with granulocyte-macrophage colony-stimulating factor (GM-CSF) and IL-4 instead of M-CSF also act as osteoclast precursors (Narisawa et al., 2021). This is particularly intriguing given that GM-CSF blockade has been explored as a potential therapeutic strategy for RA (Burmester et al., 2013; Behrens et al., 2015). The similarities and differences between AtoMs and moDCs are noteworthy because both cell types exhibit antigen-presenting cell-like properties. However, IL-4 has been reported to potently inhibit osteoclast differentiation (Lacey et al., 1995; Moreno et al., 2003), raising questions about its necessity in the differentiation of inflammatory osteoclast progenitors in RA. Rather, TNF-α, whose critical role in RA pathogenesis has been well established, may play a more important role in the differentiation of inflammatory osteoclasts. Therefore, in this study, we cultured monocytes under various conditions, including in the presence of GM-CSF with or without TNF-α, and compared their osteoclastogenic potential and bone-resorbing activity with those of conventional osteoclast precursors—namely, macrophages differentiated from monocytes in the presence of M-CSF—as well as with those of moDCs. Furthermore, we evaluated the effects of TNF-α, IL-4, and GM-CSF during the osteoclast differentiation phase induced by RANKL.

## 2 Methods

### 2.1 Cell preparation

Classical monocytes were isolated from the PBMCs of healthy volunteers using the AutoMACS cell separator (Miltenyi Biotec, Bergisch Gladbach, Germany) and Human Classical Monocyte Isolation Kit (Miltenyi Biotec), according to the manufacturer's instructions. This study was approved by the Ethical Review Committee for University Clinical Research of Jichi Medical University (approval no. CU23–056).

### 2.2 Cell culture

Cells were cultured in the α-minimum essential medium (Thermo Fisher Scientific, Waltham, MA, USA) supplemented with 10% fetal calf serum (Sigma-Aldrich Inc., St. Louis, MO, USA) and 100 U/mL penicillin with 100 μg/mL streptomycin (FUJIFILM Wako Pure Chemicals, Osaka, Japan). The cells were seeded at a density of 2.5 × 10^5^ cells/mL in the culture medium at 0.25 mL/well in 48-well culture plates (Corning Inc., Corning, NY, USA) or 48-well bone resorption assay plates (Iwasaki et al., 2024; Yamamichi et al., 2024) (Iwai Chemicals, Tokyo, Japan). The culture medium was replaced every three days. The following cytokines were used: M-CSF, GM-CSF, IL-4, and TNF-α (all from PeproTech, Cranbury, NJ, USA). For re-seeding, adherent cells were harvested using a solution of 2.5 g/L trypsin and 1 mmol/L ethylenediaminetetraacetic acid with phenol red (Nacalai Tesque Inc., Kyoto, Japan).

### 2.3 Evaluation of osteoclast generation and function

Osteoclastogenic potential and cell function were assessed using TRAP staining and pit formation assay, respectively. For TRAP staining, the cells were fixed with a neutral-buffered formalin solution (Mildform; FUJIFILM Wako Pure Chemicals), washed twice with phosphate-buffered saline, and stained using a TRAP staining kit (Cosmo Bio, Tokyo, Japan) following the manufacturer's instructions. TRAP-positive multinucleated cells (≥ 3 nuclei) were identified as osteoclasts. The number of osteoclasts was determined by counting the cells in five predefined areas within each well. For the pit formation assay, the cells were removed from the bone resorption assay plates by treatment with sodium hypochlorite for 30 s, followed by washing with distilled water. The plates were observed under an IX50 inverted microscope (Olympus, Tokyo, Japan), and images were captured using AdvanCam-HD2sP (Advan Vision, Tokyo, Japan). The pit areas were quantified using ImageJ software (version 1.54; National Institutes of Health, Bethesda, MD, USA). Measurements were taken at five distinct locations and presented as the mean ± standard deviation (SD).

### 2.4 Quantitative real-time reverse transcription PCR (qRT-PCR)

Total RNA was extracted from cultured cells using the RNeasy Plus Mini Kit (Qiagen, Venlo, Netherlands). Reverse transcription was performed using the PrimeScript II 1st strand cDNA Synthesis Kit (Takara). The resulting cDNA was diluted and amplified using TaqMan Fast Advanced Master Mix (Applied Biosystems) on a StepOnePlus Real-Time PCR System (Applied Biosystems). Relative mRNA expression levels were quantified using the ΔΔCt method. TaqMan Gene Expression Assays were used to measure the mRNA levels of *HLA-DRA1* (Hs00219575_m1), *CD80* (Hs01045161_m1), *CTSK* (Hs00166156_m1), *ITGAV* (Hs00233808_m1), *ITGB3* (Hs01001469_m1), and *GAPDH* (Hs02786624_g1) (all from Thermo Fisher Scientific). All reactions were performed in triplicate, and data are presented as the mean ± SD.

### 2.5 Statistical analysis

Statistical significance was assessed using Student's *t*-test. A *p*-value <0.05 was considered statistically significant (*: *p* < 0.05).

## 3 Results

### 3.1 Classical monocyte culture in the presence of M-CSF and RANKL

**Fig. 1:**
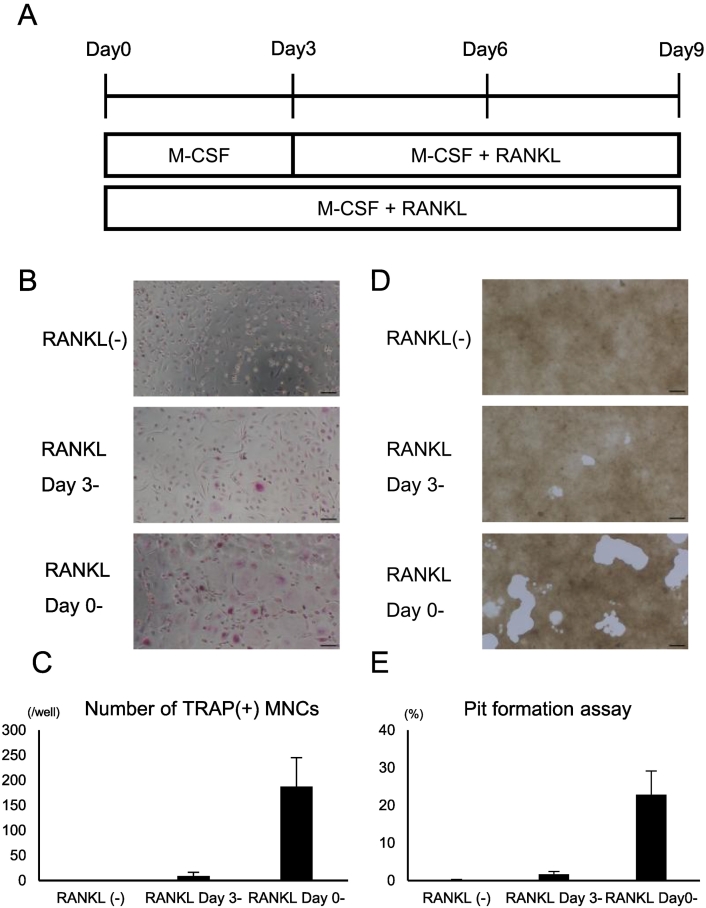
Peripheral blood-derived classical monocyte culture in the presence of macrophage colony-stimulating factor (M-CSF) and receptor activator of NF-κB ligand (RANKL). (A) Experimental scheme. Classical monocytes isolated from healthy peripheral blood were cultured with M-CSF (10 ng/mL). RANKL (50 ng/mL) was added on either day 0 or 3 of culture. (B) Representative tartrate-resistant acid phosphatase (TRAP) staining images on day 9 of culture. Scale bar, 100 μm. (C) Quantification of TRAP-positive multinucleated cells (MNCs; ≥ 3 nuclei). (D) Representative images of the resorption areas on calcium phosphate plates on day 9. Scale bar, 100 μm. (E) Quantification of the resorbed area per well in the pit formation assay. Data are represented as the mean ± standard deviation (SD) of at least three independent experiments.

First, classical monocytes derived from human peripheral blood were cultured in the presence of M-CSF, with RANKL added either from day 0 or day 3 of culture (Fig. 1A). The cells cultured with M-CSF alone differentiated into TRAP-positive mononuclear cells until day 6. In contrast, the cells exposed to RANKL formed TRAP-positive multinucleated cells with three or more nuclei (Fig. 1B). The number of TRAP-positive multinucleated cells was substantially higher when RANKL was introduced on day 0 than when it was added on day 3, and the cells were also larger (Fig. 1B and C). Furthermore, cultures on bone resorption assay plates confirmed that these cells exhibited bone-resorbing activity (Fig. 1D), with significantly greater bone resorption observed when RANKL stimulation was initiated on day 0 rather than day 3 (Fig. 1E). Thus, although pre-culture with M-CSF followed by RANKL stimulation is widely used for mouse bone marrow-derived osteoclast precursors, this step is not required for human monocytes.

### 3.2 Human osteoclast progenitor cell induction under various culture conditions

**Fig. 2:**
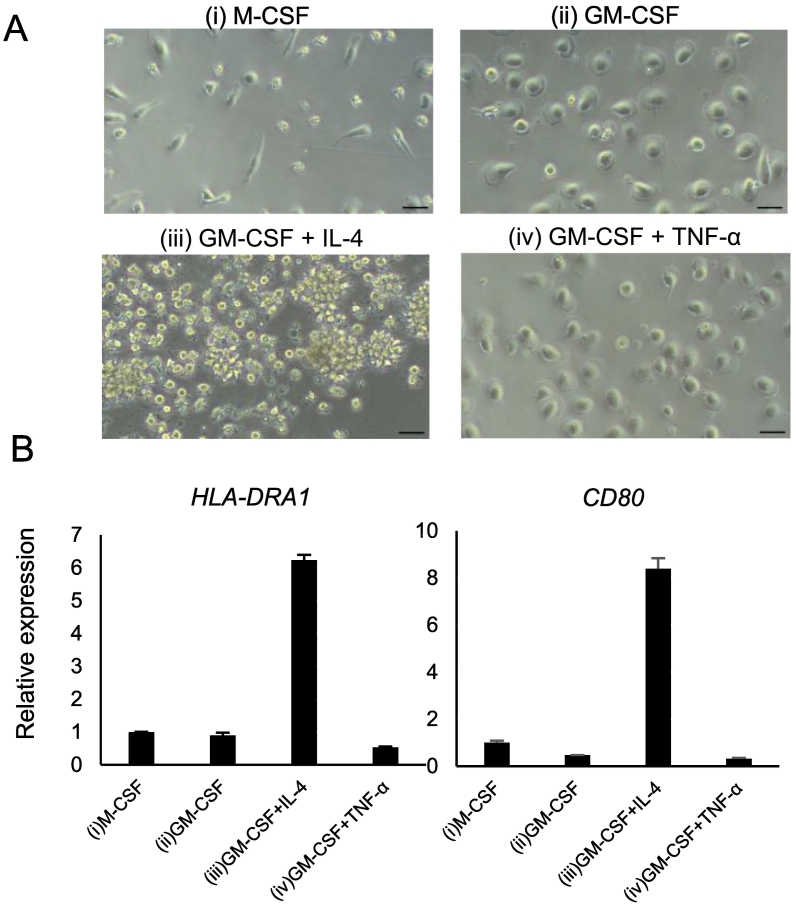
Induction of human osteoclast progenitors under various culture conditions. (A) Morphological features of human classical monocytes cultured under the following four conditions: (i) M-CSF (10 ng/mL), (ii) G-M-CSF (50 ng/mL), (iii) GM-CSF + IL-4 (50 ng/mL each), and (iv) GM-CSF (50 ng/mL) + tumor necrosis factor-alpha (TNF-α; 10 ng/mL). Scale bar: 100 μm. (B) qRT-PCR analysis of the four cell types for the expression of dendritic cell markers *HLA-DRA1* and *CD80*. Data are representative of three independent experiments.

**Fig. 3:**
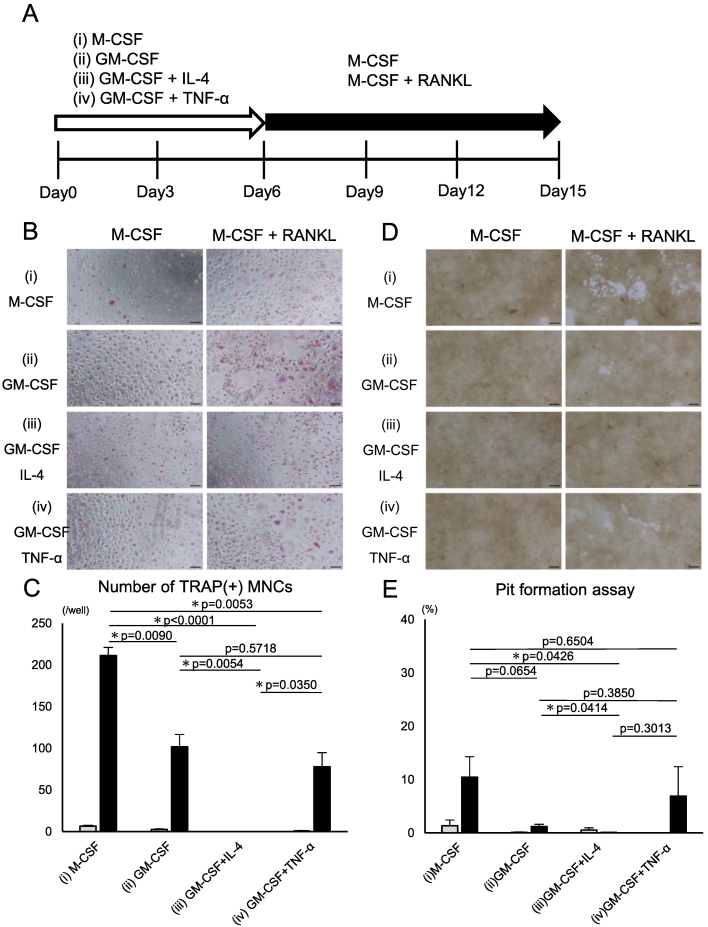

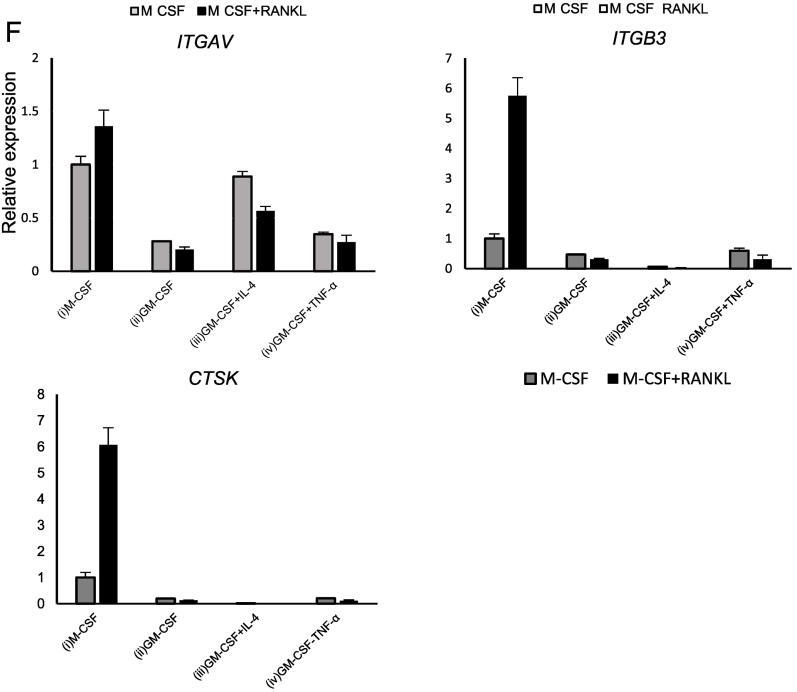
Induction of osteoclasts from various progenitors. (A) Experimental scheme. Monocytes cultured for six days under the four conditions described in Fig. 2 were subsequently cultured for an additional nine days in the presence of M-CSF (10 ng/mL) alone or M-CSF (10 ng/mL) + RANKL (50 ng/mL). (B) TRAP staining images on day 15. Scale bar: 100 μm. (C) Quantification of TRAP-positive MNCs. (D) Representative images of the resorption areas on calcium phosphate-coated plates on day 15. Scale bar: 100 μm. (E) Quantification of the resorbed areas per well in the pit formation assay. Data are represented as the mean ± SD of at least three independent experiments. (F) qRT-PCR analysis of the cells for the expression of conventional osteoclast markers integrin αvβ3 and cathepsin K. Data are representative of two independent experiments.

Next, classical monocytes were cultured for six days under the following four different conditions: (i) M-CSF, (ii) GM-CSF, (iii) GM-CSF + IL-4, and (iv) GM-CSF + TNF-α. Distinct morphological differences were observed after six days of culture, with condition (i) yielding elongated fibroblast-like cells, conditions (ii) and (iv) producing predominantly round cells, and condition (iii) yielding non-adherent floating cells (Fig. 2A). To confirm that cells cultured under condition (iii) differentiated into dendritic cells, qRT-PCR was performed. As expected, *HLA-DRA1* and *CD80* were increased at mRNA levels specifically under condition (iii) (Fig. 2B). Next, these cells were re-seeded in 48-well culture or bone resorption assay plates and cultured for nine days (Fig. 3A). Progenitor cells under conditions (i), (ii), and (iv) differentiated into TRAP-positive multinucleated cells (≥ 3 nuclei) in the presence of M-CSF and RANKL (Fig. 3B and C), whereas those under condition (iii) exhibited minimal osteoclast differentiation. Pit formation assay revealed comparable bone resorption activity under conditions (i) and (iv), weak but detectable activity under condition (ii), and no resorption activity under condition (iii; Fig. 3D and E). To assess the expression levels of the conventional osteoclast markers cathepsin K and integrin αvβ3, we performed qRT-PCR (Fig. 3F). Expression of *ITGB3*, which encodes integrin β3, and *CTSK*, which encodes cathepsin K, was markedly induced by RANKL under condition (i), but not under the other conditions. These findings indicate that progenitor cells induced by GM-CSF differentiated into cell types distinct from conventional osteoclasts, even in the presence of RANKL.

### 3.3 Osteoclast differentiation in a three-step culture system

**Fig. 4:**
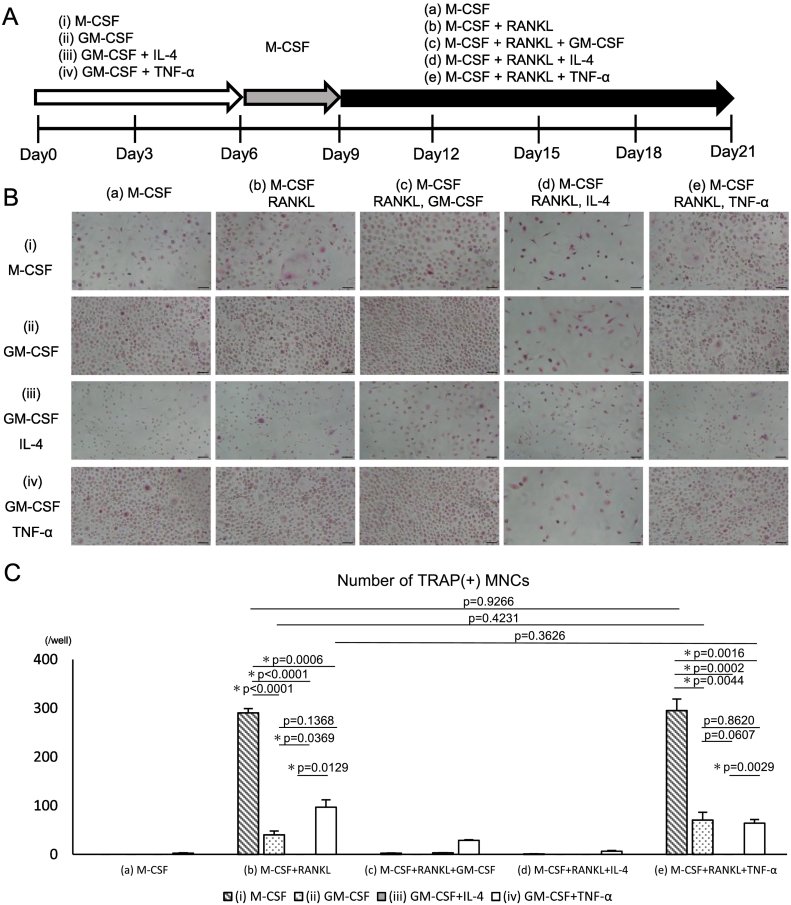
Osteoclast differentiation using a three-step culture system: TRAP staining. (A) Experimental scheme. Osteoclast progenitors were generated as shown in Fig. 3, followed by a three-day culture with M-CSF (10 ng/mL) alone. The cells were then further cultured with (a) M-CSF, (b) M-CSF + RANKL, (c) M-CSF + RANKL + GM-CSF (10 ng/mL), (d) M-CSF + RANKL + IL-4 (10 ng/mL), or (e) M-CSF + RANKL + TNF-α (10 ng/mL) for 12 d (21 days in total). M-CSF and RANKL were used at concentrations of 10 and 50 ng/mL, respectively. (B) TRAP staining images on day 21. Scale bar, 100 μm. (C) Quantification of TRAP-positive MNCs.

**Fig. 5:**
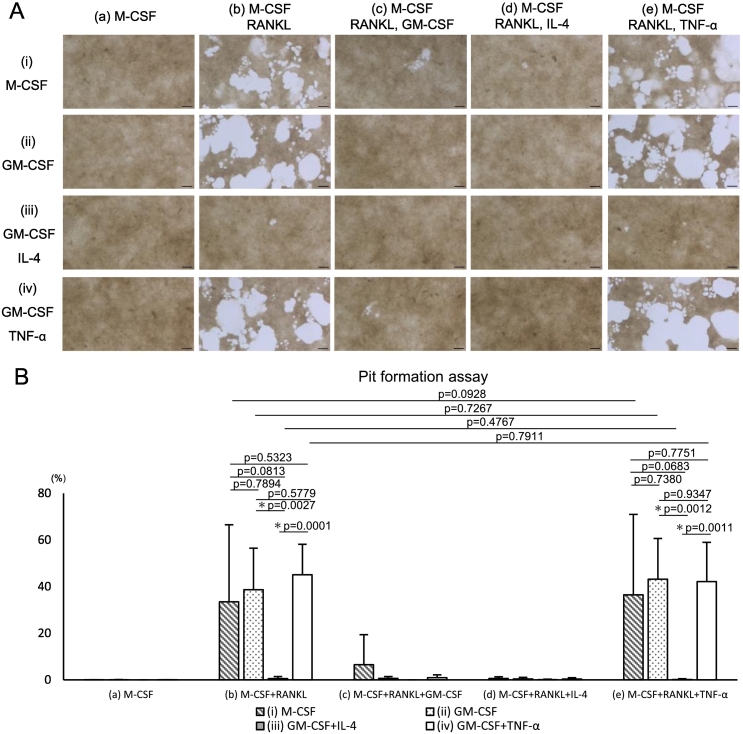
Osteoclast differentiation using a three-step culture system: Pit-formation. (A) Representative images of the resorption areas on calcium phosphate plates on day 24. Scale bar, 100 μm. (B) Quantification of the resorbed area per well in the pit formation assay. Data are represented as the mean ± SD of at least three independent experiments.

Based on the above-mentioned results, we introduced an intermediate culture step (Step 2), in which progenitor cells were cultured in 10 ng/mL M-CSF alone for three days after re-seeding (Fig. 4A). This step allowed cell adhesion under all four culture conditions, including condition (iii). Subsequently, the cells were cultured for 12 d (48-well plates) or 15 d (bone resorption assay plates) under the following conditions: (a) 10 ng/mL M-CSF, (b) 10 ng/mL M-CSF + 50 ng/mL RANKL, (c) 10 ng/mL M-CSF + 50 ng/mL RANKL +10 ng/mL GM-CSF, (d) 10 ng/mL M-CSF + 50 ng/mL RANKL +10 ng/mL IL-4, and (e) 10 ng/mL M-CSF + 50 ng/mL RANKL +10 ng/mL TNF-α. Mononuclear cells with round or elongated morphology were observed under conditions (i) and (iii) and all subsequent conditions (a–e), whereas the cells under conditions (ii) and (iv) primarily exhibited a spread circular shape. Under condition (a), in which RANKL was not added throughout the culture period, no TRAP-positive multinucleated cells were detected under conditions (i), (ii), and (iii), with only a few observed under condition (iv). Additionally, no bone resorption was detected under condition (a). When M-CSF and RANKL were added from day 9 onward (condition [b]), TRAP-positive multinucleated cells were observed under all conditions, with conditions (i), (ii), and (iv) showing higher cell numbers than condition (iii; Fig. 4B and C). Pit formation assay also confirmed bone resorption under condition (b), with the resorption area being substantially smaller under condition (iii) than under the other conditions (Fig. 5A and B). TRAP-positive multinucleated cells and resorption pits were barely observed under conditions (c: M-CSF + RANKL + GM-CSF) and (d: M-CSF + RANKL + IL-4). In contrast, under condition (e: M-CSF + RANKL + TNF-α), the number of TRAP-positive multinucleated cells and their bone-resorbing activity were comparable to those observed under condition (b). Notably, under conditions (b) and (e), the number of TRAP-positive multinucleated cells was substantially lower under conditions (ii) and (iv) compared to condition (i); however, bone-resorbing activity was comparable among conditions (i), (ii), and (iv). Overall, the number of TRAP-positive multinucleated cells and bone-resorbing activity were minimal when moDCs (condition [iii]) were used as progenitor cells (Fig. 5A and B).

## 4 Discussion

Recent studies have suggested that osteoclasts with physiological functions and those responsible for inflammatory bone destruction differ at the progenitor cell stage. In addition to AtoMs (Hasegawa et al., 2019), moDCs have been proposed as potential progenitor cells for inflammatory osteoclasts (Narisawa et al., 2021). moDCs are dendritic cells generated from monocytes cultured with GM-CSF and IL-4 (Akagawa et al., 1996). GM-CSF has been implicated in the pathogenesis of RA (Hodge et al., 2016) and its blockade has been explored as a therapeutic strategy (Burmester et al., 2013). However, a recent phase III clinical trial (CONTRAST-3) of otilimab, an anti-GM-CSF antibody, in patients with RA failed to demonstrate its significant superiority over placebo or non-inferiority to the anti-IL-6 receptor antibody, sarilumab (Taylor et al., 2023).

RA has traditionally been classified as a Th1-mediated disease based on Th1/Th2 balance (Smolen et al., 1996). Despite the subsequent discovery of Th17 cells, RA is not considered a Th2-dominant condition. Since IL-4 strongly inhibits osteoclastogenesis (Lacey et al., 1995; Moreno et al., 2003), it remains unclear whether moDCs, which require high IL-4 levels for differentiation, truly serve as inflammatory osteoclast progenitors. To verify this, we aimed in this study to explore alternative osteoclast progenitor differentiation systems independent of IL-4, specifically those using GM-CSF alone or GM-CSF combined with TNF-α. GM-CSF + TNF-α induces Langerhans cell-like dendritic cells from cord blood-derived CD34^+^ cells (Caux et al., 1992). This cytokine combination promotes dendritic cell progenitor differentiation and enhances antigen-presenting functions upon lipopolysaccharide stimulation (Iwamoto et al., 2007). As TNF-α is a key mediator in RA pathogenesis and anti-TNF therapies have greatly advanced RA treatment (Breedveld and Combe, 2011), progenitors generated under GM-CSF + TNF-α conditions may also function as osteoclast precursors. Classical monocytes cultured with GM-CSF alone exhibit a morphology distinct from that of monocytes cultured with M-CSF alone. The former, referred to as “GM-CSF-type macrophages,” predominantly displayed a round cell shape, whereas the latter, “M-CSF-type macrophages,” exhibited a spindle-shaped morphology (Fig. 2A). GM-CSF- and M-CSF-type macrophages have also been categorized as M1-and M2-type macrophages, respectively (Verreck et al., 2004). Conventional osteoclast precursors can be regarded as macrophages induced in the presence of M-CSF, namely M2-type macrophages.

Interestingly, moDCs exhibited negligible TRAP-positive multinucleated cell formation and bone-resorbing activity when cultured with M-CSF and RANKL (Fig. 3B-3E). Although this appears to contradict the previous report, we observed that moDCs remained non-adherent, floating in culture, unlike cells induced under other conditions. Using qRT-PCR, we evaluated the mRNA expression levels of conventional osteoclast differentiation markers, integrin αvβ3 and cathepsin K. Progenitor cells induced in the presence of GM-CSF did not show upregulation of these markers even in the presence of RANKL, suggesting that they belong to distinct lineages from conventional osteoclasts (Fig. 3F).

As cell adhesion is a prerequisite for osteoclast formation (Miyamoto et al., 2000), we introduced an intermediate M-CSF-only culture step (Step 2) between progenitor cell induction and osteoclast differentiation steps to establish a three-step culture system (Fig. 3). Floating moDCs, when exposed to M-CSF alone, transformed into spindle-shaped adherent cells resembling the M2-type macrophages, rather than the M1-type macrophages. This finding is consistent with observations by Akagawa and colleagues (Akagawa et al., 1996), who demonstrated that moDCs cultured in the absence of GM-CSF but in the presence of M-CSF re-differentiate into M2-type macrophages. When osteoclast differentiation was induced in the third step, the moDC-derived cells showed TRAP-positive multinucleated cell formation and bone-resorbing activity. The M-CSF culture step (Step 2) was also described by Narisawa et al. During this step, moDCs may undergo phenotypic changes; however, Narisawa et al. demonstrated that even after differentiation into osteoclasts, these cells retain dendritic cell-like properties such as the expression of CD80, CD86, and HLA-DR. Nevertheless, the differentiation efficiency and bone-resorbing activity of the moDCs did not match those of progenitor cells induced under other conditions. Although Narisawa et al. demonstrated the superiority of moDC-derived osteoclasts over monocyte-derived osteoclasts (Narisawa et al., 2021), they used ivory sections for evaluation, in contrast to our study, which used bone resorption assay plates. Such minor differences in methodology may explain the observed discrepancies.

We also found that the addition of IL-4 and GM-CSF during the osteoclast differentiation phase (Step 3) inhibited both osteoclast differentiation and bone-resorbing activity, even at lower concentrations (10 ng/mL) than those used for moDC generation (50 ng/mL in Step 1). This observation aligns with previous reports (Udagawa, 2003; Moreno et al., 2003). In contrast, the same concentration of TNF-α did not impair osteoclast differentiation or function. Akagawa et al. also reported that moDCs undergo apoptosis upon TNF-α stimulation, even in the presence of M-CSF, attributing this to suppressed expression of M-CSF receptor (c-fms). As TNF-α is supposed to be abundant in the RA-affected synovial microenvironment, moDCs may have limited survival potential under such inflammatory conditions. Therefore, dendritic cell progenitors induced by GM-CSF + TNF-α (Iwamoto et al., 2007) and GM-CSF-type macrophages may be more suitable as inflammatory osteoclast progenitors than moDCs. In any case, whether unconventional osteoclasts derived from these cells or from moDCs are actually present in the synovium of patients with RA, and if so, how frequently they occur, remains an important question to be addressed in future studies.

This study attempted to recapitulate RA-associated osteoclast differentiation in vitro. However, the actual inflammatory joint environment in patients with RA is highly complex. Unlike the relatively simplified culture conditions used in the present study, synovial fibroblasts and various immune cells may interact with progenitor cells via numerous humoral and adhesive factors, the full scope of which remains poorly understood. For example, other proinflammatory cytokines, such as IL-6 and IL-1, have been implicated in the pathogenesis of RA. In addition, interferon-γ, which is produced by T cells in RA joint fluid (Smolen et al., 1996; Yamada et al., 2008), strongly suppresses osteoclastogenesis (Takayanagi et al., 2000; Sato et al., 2006). Nevertheless, osteoclasts are present in the synovium of RA patients and actively contribute to bone destruction. If these cytokines were added to the in vitro culture system used in this study, the number of experimental conditions would increase exponentially, rendering it technically unfeasible to conduct experiments using human primary cells. Future studies should aim to establish a simplified yet representative in vitro culture system—a “toy model.” As a subsequent step, this model could be made more complex and physiologically relevant by incorporating patient-derived samples from individuals with inflammatory joint diseases such as RA, or by introducing synovial fibroblasts to create a co-culture system. Such enhancements are expected to deepen our understanding of RA pathogenesis and contribute to the development of novel therapeutic strategies.

## CRediT authorship contribution statement

**Shiho Kinoshita:** Writing – original draft, Visualization, Investigation. **Yasuhiro Omata:** Validation, Investigation. **Kojiro Sato:** Writing – review & editing, Supervision, Project administration, Methodology, Funding acquisition, Conceptualization.

## Funding statement

This study was partially supported by Grants-in-Aid for Scientific Research from the 10.13039/501100001691Japan Society for the Promotion of Science (21 K09284 and 24 K11602).

## Declaration of competing interest

The authors declare that they have no known competing financial interests or personal relationships that could have appeared to influence the work reported in this paper.

## Data Availability

Data will be made available on request.
